# Supplementary material for: Astrocyte and L-lactate in the anterior cingulate cortex modulate schema memory and neuronal mitochondrial biogenesis
Source: eLife. 2023 Nov 14;12:e85751. doi: 10.7554/eLife.85751 (PMC10645423; doi:10.7554/eLife.85751)
Supplement: Figure 3—source data 1. [file elife-85751-fig3-data1.zip › Supplementary File 1.docx]

### Supplementary File 1. Comparison of performance index of control vs. hM4D_i_-CNO group

(Unpaired t test, FDR (5%) correction with two-stage step-up method of Benjamini, Krieger and Yekutieli)

| **Sessions** | **P value** | **Mean PI of hM4D_i_-CNO group** | **Mean PI of control group** | **Difference** | **SE of difference** | **t ratio** | **df** | **q value** |
| --- | --- | --- | --- | --- | --- | --- | --- | --- |
| S1 | 0.132910 | 33.56 | 37.50 | -3.944 | 2.523 | 1.563 | 21 | 0.008722 |
| S2 | 0.028104 | 37.78 | 44.17 | -6.389 | 2.709 | 2.359 | 21 | 0.002108 |
| S4 | 0.002756 | 40.67 | 49.58 | -8.917 | 2.630 | 3.391 | 21 | 0.000223 |
| S5 | 0.030648 | 44.22 | 50.83 | -6.611 | 2.853 | 2.318 | 21 | 0.002145 |
| S6 | 0.000011 | 49.11 | 62.50 | -13.39 | 2.337 | 5.730 | 21 | 0.000002 |
| S7 | 0.000013 | 48.44 | 64.58 | -16.14 | 2.858 | 5.646 | 21 | 0.000002 |
| S8 | <0.000001 | 52.00 | 69.58 | -17.58 | 2.263 | 7.770 | 21 | <0.000001 |
| S10 | 0.000014 | 52.00 | 64.58 | -12.58 | 2.234 | 5.631 | 21 | 0.000002 |
| S11 | 0.000005 | 53.78 | 67.92 | -14.14 | 2.315 | 6.109 | 21 | <0.000001 |
| S12 | 0.000119 | 55.33 | 68.33 | -13.00 | 2.760 | 4.711 | 21 | 0.000010 |
| S13 | 0.000109 | 57.56 | 70.83 | -13.28 | 2.797 | 4.747 | 21 | 0.000010 |
| S14 | 0.000001 | 53.78 | 73.89 | -20.11 | 3.037 | 6.622 | 21 | <0.000001 |
| S15 | 0.000020 | 58.00 | 72.38 | -14.38 | 2.627 | 5.475 | 21 | 0.000002 |
| S16 | <0.000001 | 59.11 | 77.14 | -18.03 | 2.471 | 7.298 | 21 | <0.000001 |
| S17 | <0.000001 | 61.25 | 77.62 | -16.37 | 2.133 | 7.675 | 21 | <0.000001 |
| S19 | 0.000005 | 50.22 | 69.52 | -19.30 | 3.186 | 6.059 | 21 | <0.000001 |
