# Supplementary material for: Astrocyte and L-lactate in the anterior cingulate cortex modulate schema memory and neuronal mitochondrial biogenesis
Source: eLife. 2023 Nov 14;12:e85751. doi: 10.7554/eLife.85751 (PMC10645423; doi:10.7554/eLife.85751)
Supplement: Supplementary file 2. [file elife-85751-supp2.docx]

### Supplementary File 2. Comparison of performance index of control vs. rescue group

(Unpaired t test, FDR (5%) correction with two-stage step-up method of Benjamini, Krieger and Yekutieli)

| **Sessions** | **P value** | **Mean PI of control group** | **Mean PI of Rescue group** | **Difference** | **SE of difference** | **t ratio** | **df** | **q value** |
| --- | --- | --- | --- | --- | --- | --- | --- | --- |
| S1 | 0.280073 | 37.50 | 35.56 | 1.944 | 1.725 | 1.127 | 13 | 0.382300 |
| S2 | 0.489187 | 44.17 | 41.90 | 2.262 | 3.178 | 0.7118 | 13 | 0.547945 |
| S4 | 0.198017 | 49.58 | 46.19 | 3.393 | 2.501 | 1.357 | 13 | 0.300326 |
| S5 | 0.074493 | 50.83 | 58.10 | -7.262 | 3.745 | 1.939 | 13 | 0.168616 |
| S6 | 0.512548 | 62.50 | 64.29 | -1.786 | 2.652 | 0.6733 | 13 | 0.547945 |
| S7 | 0.521852 | 64.58 | 67.14 | -2.560 | 3.888 | 0.6583 | 13 | 0.547945 |
| S8 | 0.019799 | 69.58 | 76.19 | -6.607 | 2.488 | 2.656 | 13 | 0.055388 |
| S10 | 0.000193 | 64.58 | 77.50 | -12.92 | 2.517 | 5.132 | 13 | 0.002630 |
| S11 | 0.003092 | 67.92 | 77.14 | -9.226 | 2.546 | 3.623 | 13 | 0.014068 |
| S12 | 0.003050 | 68.33 | 81.85 | -13.52 | 3.724 | 3.630 | 13 | 0.014068 |
| S13 | 0.172378 | 70.83 | 74.76 | -3.929 | 2.720 | 1.444 | 13 | 0.294120 |
| S14 | 0.086470 | 73.89 | 79.05 | -5.159 | 2.782 | 1.855 | 13 | 0.168616 |
| S15 | 0.020289 | 72.38 | 79.05 | -6.667 | 2.523 | 2.643 | 13 | 0.055388 |
| S16 | 0.653871 | 77.14 | 78.57 | -1.429 | 3.113 | 0.4589 | 13 | 0.600791 |
| S17 | 0.677042 | 77.62 | 78.57 | -0.9524 | 2.235 | 0.4260 | 13 | 0.600791 |
| S19 | 0.704224 | 69.52 | 68.10 | 1.429 | 3.681 | 0.3881 | 13 | 0.600791 |
