# Supplementary material for: Astrocyte and L-lactate in the anterior cingulate cortex modulate schema memory and neuronal mitochondrial biogenesis
Source: eLife. 2023 Nov 14;12:e85751. doi: 10.7554/eLife.85751 (PMC10645423; doi:10.7554/eLife.85751)
Supplement: Supplementary file 3. [file elife-85751-supp3.docx]

### Supplementary File 3. Primer sequences and preparation of 20 μl reaction mixture for real-time PCR

| **Component with concentration** | **Volume added** | **Final concentration/ amount** |
| --- | --- | --- |
| 2X, SsoAdvanced Universal SYBR Green Supermix, (Bio-Rad) | 10 μl | 1X |
| 5 ng/μL genomic DNA | 2 μl | 10 ng |
| Rat D-loop Forward Primer: 5′-GGTTCTTACTTCAGGGCCATCA-3′ (5 μM)  [For mtDNA reaction] | 2 μl | 400 nM |
| Rat D-loop Reverse Primer: 5′-GATTAGACCCGTTACCATCGAGAT-3′ (5 μM)  [For mtDNA reaction] | 2 μl | 400 nM |
| Rat β-actin Forward Primer: 5′-GGGATGTTTGCTCCAACCAA-3′ (5 μM)  [For nDNA reaction] | 2 μl | 400 nM |
| Rat β-actin Reverse Primer: 5′-GCGCTTTTGACTCAAGGATTTAA-3′ (5 μM)  [For nDNA reaction] | 2 μl | 400 nM |
| Nuclease free H_2_0 | 4 μl |  |
